# A Case of Blastomyces dermatitidis Diagnosed Following Travel to Colorado: A Case Report and Review of Literature

**DOI:** 10.7759/cureus.44733

**Published:** 2023-09-05

**Authors:** Gaurav Venkat Cuddapah, Mayra Z Malik, Amulya Arremsetty, Andrew Fagelman

**Affiliations:** 1 Internal Medicine, Kamineni Academy of Medical Sciences and Research Centre, Secunderabad, IND; 2 Internal Medicine, CMH Lahore Medical College and Institute of Dentistry, New York, USA; 3 Internal Medicine, Siddhartha Medical College, Secunderabad, IND; 4 Internal Medicine, NYU Grossman School of Medicine (NYU Langone Health), New York, USA

**Keywords:** fungal culture, itraconazole, new york city, pneumonia, blastomyces dermatitidis

## Abstract

The dimorphic fungus *Blastomyces dermatitidis*, is one of the most frequent causes of endemic fungal infections in the United States as well as various other parts of the world. Clinical presentations vary widely, ranging from asymptomatic to disseminated systemic infections. Blastomycosis usually has a predilection for the lungs, but extra pulmonary manifestations are present in 25-40% of cases, involving the skin, bone, genitourinary tract, and CNS. A fungal culture of tissue specimens and fluids is confirmatory. The mainstay of treatment are the azole antifungals, i.e., itraconazole, and for disseminated disease, amphotericin B. We present a case of a young male with pulmonary blastomycosis who presented with a long incubation period. The non-resolving nature of his symptoms prompted further lab and imaging studies, ultimately leading to full and successful recovery.

## Introduction

*Blastomyces dermatitidis* is a dimorphic fungus that is found as a yeast at body temperature, it resides within the soil as spores. The conidia becomes aerosolised and when inhaled enters the lungs resulting in a pulmonary infection. Infected tissue samples will yield the thick- walled yeast form of the *Blastomyces dermatitidis*, which confers its resistance to the immune system. Blastomyces is historically endemic to Eastern, South Eastern, Central and the Great Lakes region of the United States. Blastomycosis can however be sporadic. The 2007 to 2017 data from New York revealed 0.1 to 0.2 cases per 100,000. Globally speaking most cases of blastomycosis arise from Africa as well as South America, India, China and the Middle East [1]. This infection has shown more prevalence in males likely associated with occupational exposure. The immunocompetent usually remain asymptomatic with complete recovery. However, the immunocompromised have poorer prognosis. The immune defence against Blastomyces involves adequate T-lymphocytes function and a loss of it as in HIV, carcinomas or any organ transplantation can lead to more severe and disseminated disease [2]. The lung is the primary target of infection and extra pulmonary disease can occur in a smaller percentage of the population. Blastomycosis can present with a vast array of symptoms and a strong clinical suspicion is needed to arrive at the correct diagnosis. It can masquerade as for example the common cold or flu like illness or even Tuberculosis in the more underdeveloped parts of the world. The initial presentation could be a flu-like illness with fever, chills, rigors, and non- productive cough that subsides within days. Because of the self-limited course of the initial presentation, it may go undiagnosed. The fungal pneumonia can present with high fever, a productive cough and pleuritic chest pain mimicking bacterial pneumonia. Extra pulmonary manifestations can involve the skin as subcutaneous nodules, the bone with joint pain and arthritis, the genito-urinary tract as prostatitis and the CNS as meningitis [3]. Lung involvement can lead to consolidation with or without pleural effusion but radiographic imaging lacks diagnostic accuracy. Therefore, the gold standard for diagnosis is fungal cultures and histopathology to identify the broad-based budding yeast forms under microscopy. In the differential count, leukocytosis with a left shift is commonly seen especially with lung manifestations [4]. The incubation period is typically three weeks to three months, but our case exhibited a longer incubation period of four months before the onset of symptoms. His initial presentation was vague mimicking the common cold, and he later presented with a chronic productive cough with gradual progression. We hereby demonstrate a case of a 36-year-old male who traveled to Colorado, which is traditionally a non-endemic region of the United States. He participated in various outdoor activities including hiking, and spent a majority of his time exploring the woods. He presented after an unusually longer incubation period with a clinical presentation too ambiguous for a prompt diagnosis. Despite trials of empiric antibiotics for a presumed lung infection, he did not recover until further testing revealed *Blastomyces dermatitidis*. We learned from this case that a travel history to even a non-endemic region should still raise a high index of clinical suspicion for *Blastomyces dermatitidis*. Treatment is with the azole antifungals for pulmonary symptoms and amphotericin B for disseminated manifestations [3].

## Case presentation

**Figure 1:**
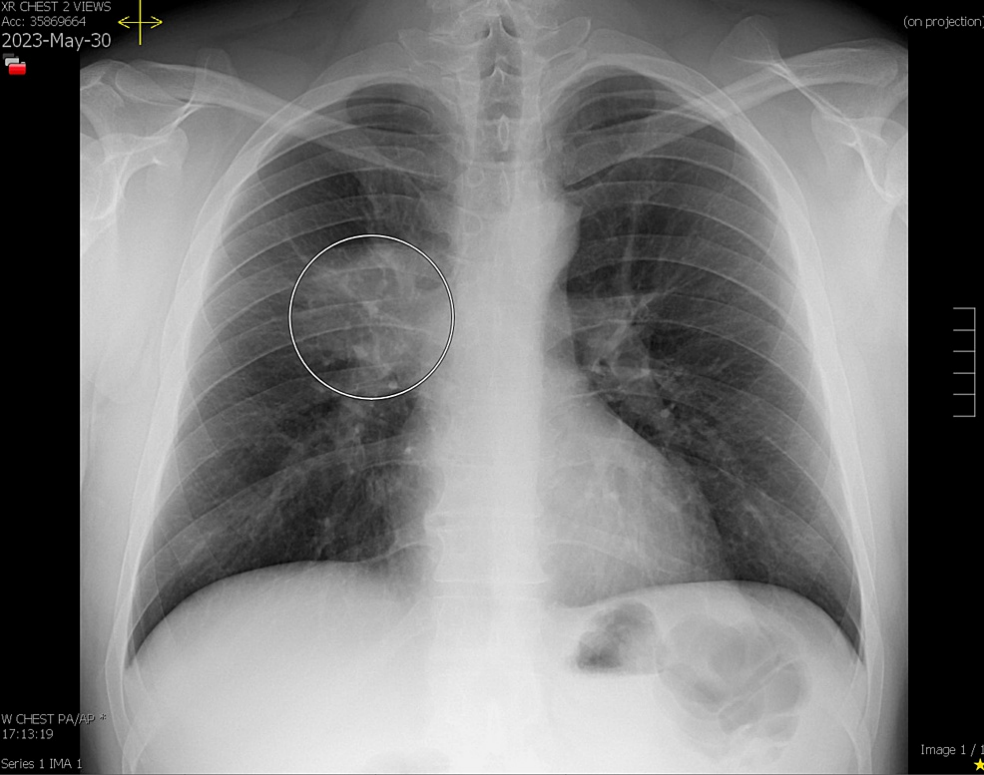
Chest X-ray depicting right upper lobe consolidation (posteroanterior view)

**Figure 2:**
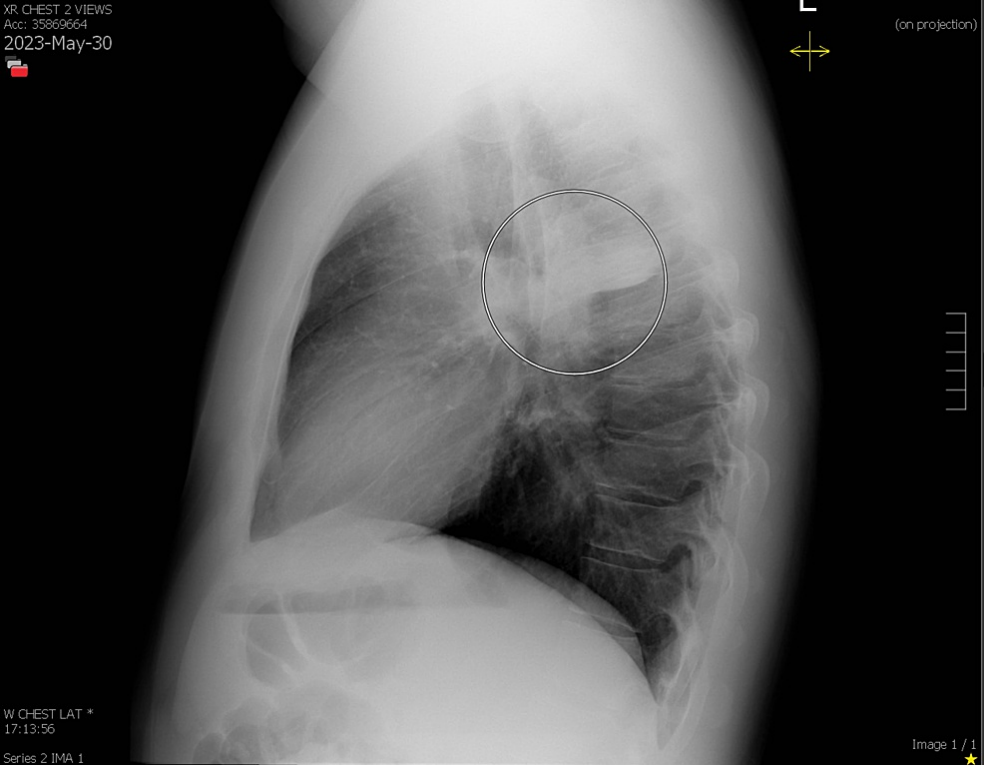
Chest X-ray depicting right upper lobe consolidation (lateral view)

In January of 2023, a 36-year-old man presented to the clinic in New York City with complaints of a runny nose and congestion for two days. No nausea, vomiting, fever, chest pain, or shortness of breath were reported. Prior to the onset of symptoms, he did have a history of travel to Colorado in December. In Colorado, of noteworthy was his hiking, and he was exposed to the woods and vegetable matter. The results of a test for COVID-19 were negative. He had no past medical or surgical history of significance. He had never been a smoker. Antihistamines, nasal mists, and cough syrups were recommended over-the-counter. In the subsequent four months, he experienced a significant improvement in his symptoms and became asymptomatic. Early May, he returned to the clinic with a cough that deteriorated in the evening for a week. A white sputum accompanied the wheeze. His symptoms worsened progressively, but there was no fever, and a repeat COVID test was negative. A seven-day course of azithromycin 250 mg and prednisone was prescribed. At the end of May, he returned to the clinic with a persistent cough that produced clear sputum. Antibiotics were administered again, this time doxycycline 100 mg twice daily for seven days. A chest X-ray examination revealed consolidation of the right upper lobe (Figures 1, 2) .

**Figure 3:**
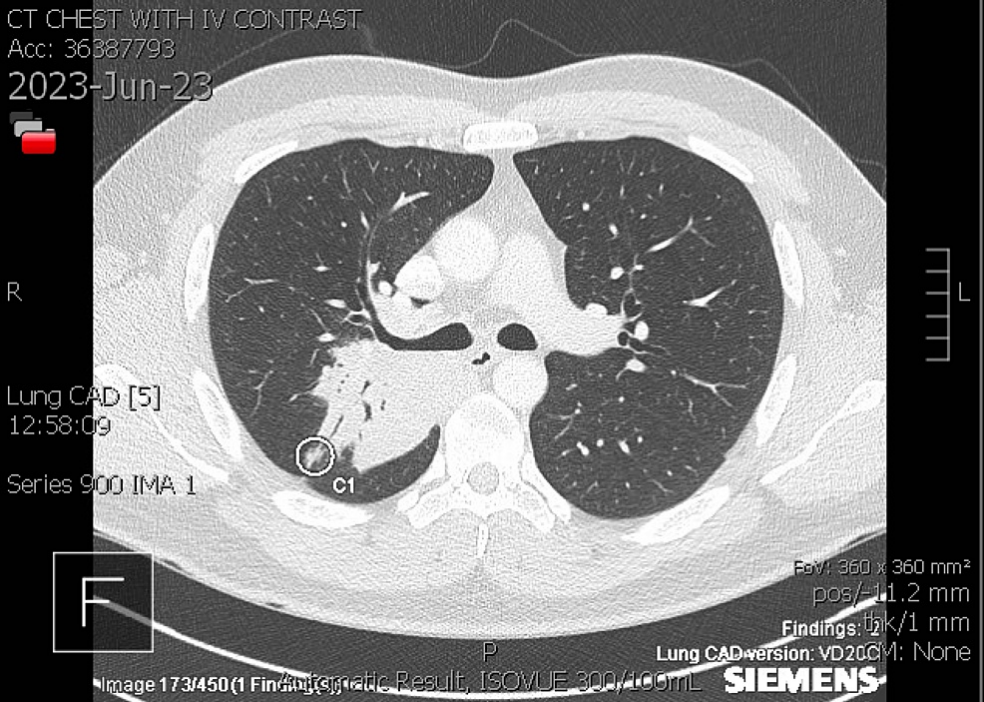
CT Scan of the chest with IV contrast depicting air bronchograms and right upper lobe posterior segment mass (axial view)

**Figure 4:**
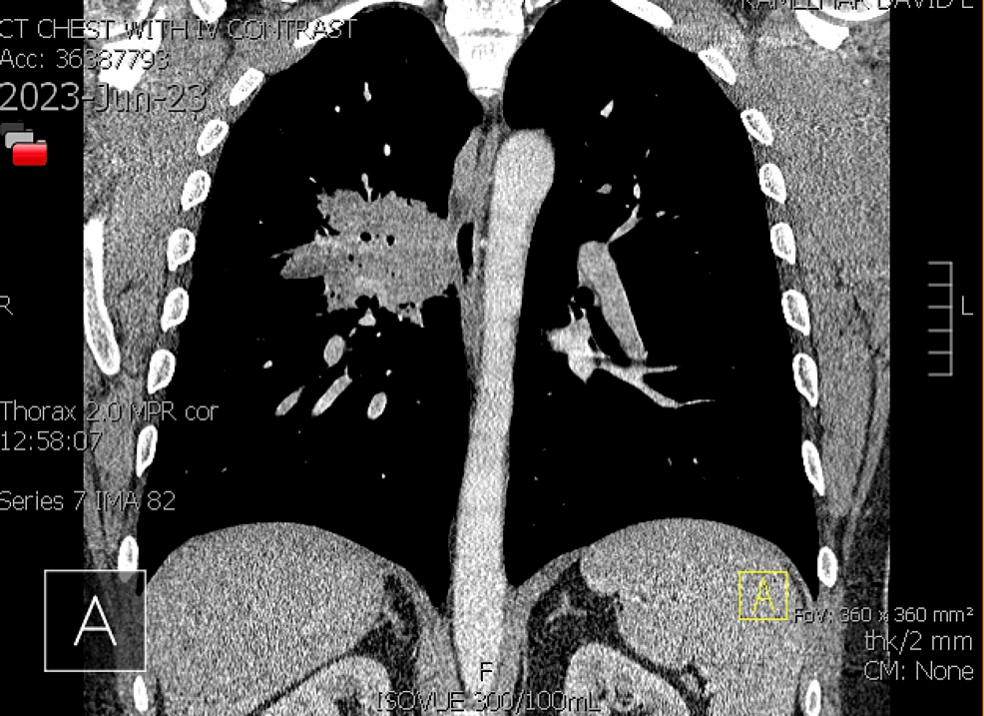
CT chest with IV contrast showing a lobulated right upper lobe mass (coronal view)

**Figure 5:**
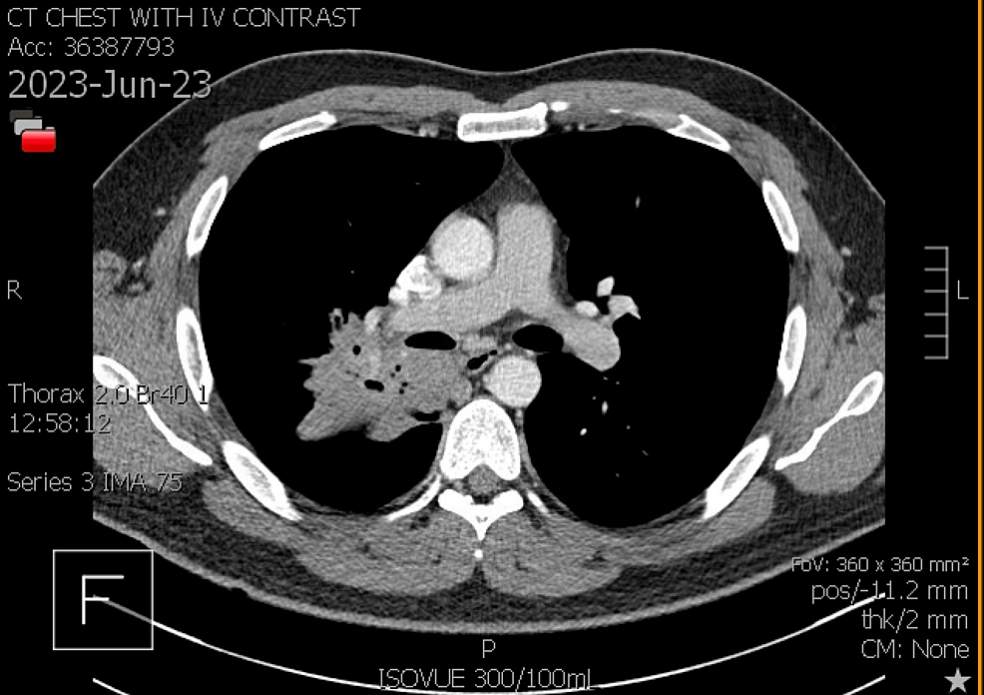
CT chest with IV contrast clearly showing a mass in the right upper lobe posterior segment (axial view)

By mid-June, he returned for a follow-up visit with no improvement from the antibiotics. The productive cough was now accompanied by brown sputum in the morning and white sputum throughout the day. Corticosteroids administered did not completely alleviate symptoms. We considered infection, postnasal drip, reflux, and asthma as possible diagnoses at the time. A trial course of advair, flonase, zyrtec D, and famotidine 20 mg was prescribed to the patient. He was referred to the pulmonologist for additional evaluation. The patient's symptoms worsened at the end of June. Lying down exacerbated the cough, which was accompanied by fever, dark red sputum, and right-sided pleuritic chest discomfort. CT chest with IV contrast was notable for a large consolidation in the right upper lobe posterior segment, with lobulated contours and air bronchograms. In addition, right lower lobe superior segment and mild involvement of the right middle lobe was also seen (Figures 3, 4, 5).

The following shows the summary of the patient’s pertinent lab findings (Table 1).

**Table 1:** Pertinent lab findings ESR: Erythrocyte sedimentation rate; CRP: C reactive protein

	Temperature (°F)	White Blood Cell Count	Lymphocyte Count	Neutrophil Count	ESR	CRP
01/03/23	97.3°F	7.2 10^3^/uL	2.2 10^3^/uL	4.0 10^3^/uL	-	-
06/28/23	98.8°F	11.3 10^3^/uL	1.2 10^3^/uL	9.0 10^3^/uL	-	-
07/11/23	100.1°F	12.1 10^3^/uL	1.3 10^3^/uL	9.6 10^3^/uL	73	21.6

**Figure 6:**
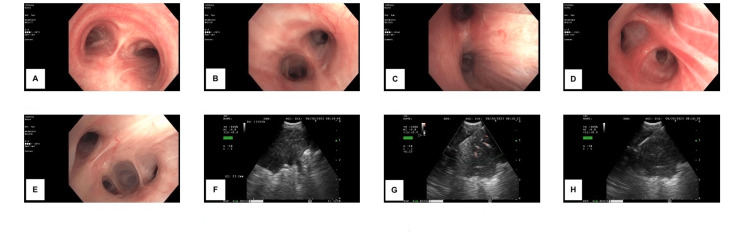
Bronchoscopy showing right upper lobe erythema, edema and a heterogenous mass in the posterior segment right upper lobe A: Trachea; B: Left mainstem bronchus; C: Right mainstem bronchus; D: Right upper lobe; E: Bronchus intermedius; F: Right upper lobe lesion; G: Right upper lobe lesion biopsy; H: Right upper lobe lesion biopsy

Bronchoscopy performed at the end of June revealed right upper lobe erythema, edema, and a heterogeneous mass in the posterior segment of the right upper lobe (Figure 6). A biopsy sample was collected. By immunodiffusion, anomalous serum Blastomyces antibodies and *Blastomyces dermatitidis* antigens were detected in urine on July 11. Other differential diagnoses were considered including antibodies for HIV, histoplasma, and sputum analysis for tuberculosis. The fungal culture of bronchoalveolar lavage grown for 28 days was positive for moderate *Blastomyces dermatitidis* on July 27, as was the fungal culture of tissue with sabouraud dextrose agar. 

The patient was administered 100 mg of itraconazole once daily for six months. During the first week of treatment, a quantitative serum test for itraconazole ensured adequate therapeutic levels of 1.0 ug/mL (therapeutic range ≥ 0.6). The patient's symptoms of cough with productive sputum improved at his subsequent follow-up appointment, thus proving response to treatment. 

## Discussion

Blastomycosis is a systemic granulomatous infection caused by the inhalation of conidia from the thermally dimorphic fungi *Blastomyces dermatitidis* or *Blastomyces gilchristii* found in the soil. Conidia endure nonspecific phagocytosis and lysis mediated by alveolar macrophages, polymorphonuclear leukocytes (PMN) and monocytes following inhalation, resulting in an asymptomatic course in a minority of individuals. Consequently, the lungs are the most prevalent infection site [5]. It can cause symptomatic lung infection when *Blastomyces dermatitidis* transforms into the yeast form because of the thick wall's ability to resist phagocytosis and destruction. Additionally, the immune-modulating glycoprotein BAD-1 makes it easier for it to connect to macrophages, allowing it to spread to other parts of the body via the blood and lymphatic system. A distinct characteristic of blastomycosis is the pyogranulomatous inflammatory response, which is brought on by an influx of neutrophils and macrophages and ultimately results in the formation of granulomas [1].

*Blastomyces dermatitidis* is traditionally associated with the central United States. The states bordering the Great Lakes, as well as the Ohio and Mississippi River valleys, are geographically distributed. It most frequently manifests as an asymptomatic and therefore undetectable pulmonary infection, although life-threatening complications such as acute respiratory distress syndrome may occur. Pulmonary blastomycosis can be accompanied by various other rare findings like cutaneous lesions, pleural effusion, and brain abscess. Studies by Alvarez et al. on a young African man who presented with pleural effusion and Slomka et al. on a rare case of *Blastomyces dermatitidis *brain abscess in an immunocompetent patient have demonstrated the infrequent presentation of Blastomyces [6,7]. 

Although our patient had a long asymptomatic period of about four months, there have been cases of symptomatic *Blastomyces dermatitidis* in Colorado. Groote et al. demonstrated two such cases [8]. In both cases, the symptoms of cough, fever, chest pain, and arthralgia were prominent features for approximately two weeks before admission to the hospital. These individuals were exposed to the fungus through occupational work, digging matter in an area infested with species including rodents and bats. Similar to our patient, these cases presented with predominantly pulmonary symptoms and thus were treated empirically for community acquired pneumonia or bronchitis. Also, similar to our patient, the modes used for diagnosis, after excluding possibilities like HIV, were chest X ray, CT chest, DNA probe, lung biopsies, and fungal cultures [8].

After hematogenous spread from the lungs, nearly 25% to 30% of patients develop extrapulmonary disease. Despite its rarity, primary cutaneous blastomycosis can be caused by direct inoculation following skin trauma. In contrast to other profound fungal infections that primarily affect immunocompromised patients, blastomycosis can also affect immunocompetent hosts [1]. Our patient, like the instance described by Wang et al., had visited areas of the United States where the fungus is endemic [5]. However, unlike the patient in the prior imported case, who had a chronic cough after exposure, our patient was initially symptomatic for a week, which subsided on medication, and was completely asymptomatic for the next four months, with no predisposing condition.

Our patient presented atypically, as he did not develop a fever until late in the course. After an initial asymptomatic period and resolution of cough with the use of inhalational corticosteroids, we considered a number of alternative diagnoses, including cough variant asthma and tuberculosis. The plausible causes for a delay in symptom onset include the use of over-the-counter medications during the initial episode of runny nose and congestion may have suppressed the body's symptomatic response to the fungi, the patient's high immunity and the use of systemic corticosteroids during the second phase of symptoms may have suppressed the immune system, thereby increasing the virulence of the fungus and leading to the onset of symptoms.

Only when he presented with blood in his sputum and his chest X-ray revealed consolidation did we become alarmed, prompting us to conduct additional tests, including a chest CT, fungal antigens in his urine, and fungal antibodies in his blood. Following the results of his CT scan and positive Blastomyces antigen and antibody tests, he underwent a bronchoscopy for bronchoalveolar lavage and tissue culture, microscopy, and histopathology which turned out to be positive for *Blastomyces dermatitidis* after three weeks of incubation. Typically, yeast cells exhibit a single, broad-based budding that can be seen using the periodic acid-Schiff (PAS) or Gomori methamine silver (GMS) stains. Complex instances typically lend themselves to more specialized nucleic acid detection techniques, such as repetitive DNA sequences like next generation sequencing and polymerase chain reaction (PCR) [5]. The most definitive technique for diagnosing blastomycosis is culture. Typically, growth is detected within five to 10 days, but it may take as long as 30 days if the sample contains few organisms [1].

The study by Bennie Ho et al. demonstrated a patient with blastomycosis whose bronchial washings were negative for fungal culture after four weeks of incubation, thus demonstrating that a negative culture does not unequivocally exclude the presence of systemic *Blastomyces dermatitidis* [9]. Although spontaneous remission is possible, it is advised that all patients with mild or moderate disease receive treatment to prevent disease spread and recurrence. Itraconazole is the preferred treatment for all forms of the disease, excluding those that are fatally severe. It is suggested that 600 mg be administered every day for three days, then followed by 200 mg to 400 mg daily for six to 12 months. Amphotericin B is administered at a high dose of 0.6 to 1 mg/kg/day, for a total dose of 1.5 to 2.0 grams, for severe and life-threatening diseases. Amphotericin B at a dose of 3 to 5 mg/kg per day is an alternative treatment for severe infections and is preferred for the treatment of CNS blastomycosis and expectant women. The lipid formulation of amphotericin has been associated with adverse effects, including renal impairment [1].

Our patient’s case is unique for fungal pneumonia in that the only factor he had was travel to Colorado, an area of the United States not typically known to have endemicity to *Blastomyces dermatitidis*. Therefore, his intractable pneumonia did not meet the list of differentials diagnoses for a fungal pathogen, until further testing warranted the need for a tissue specimen. However, fungal cultures of the samples taken took did not reveal even moderate growth until 28 days later. For this patient, prompt diagnosis and treatment were made possible by the use of quicker tests, such as blood antibodies to Blastomyces.

We reviewed 14 cases of blastomycosis in various locations and summarized their characteristics (Table 2).

**Table 2:** Summary of the characteristics of patients with blastomycosis PET: Positron emission tomography

Author	Age	Gender	Location	Travel Hx	S/S	Dx	Tx
Groote et al. [8]	25 years old	Male	Colorado	None	Fever, malaise, weight loss, joint aches, cough	Chest X ray, CT chest, lung biopsy, fungal culture, DNA probe	IV amphotericin B 550 mg and oral itraconazole 400 mg/d x 5 months
Groote et al. [8]	35 years old	Male	Colorado	None	Fever, cough, chest pain, shortness of breath, fatigue, skin lesions, athralgia	Chest X ray, biopsy of skin lesions, CT chest, lung biopsy	IV amphotericin B 350 mg x 14 days, then oral itraconaozle 400 mg/d x 6 months
Wang et al. [5]	24 years old	Male	China	Illinois, USA	Recurrent non-productive cough with white sputum	Chest X ray, CT chest, bronchoscopy, endobronchial ultrasound with transbronchial lung biopsy, bronchoalveolar lavage fluid, nasogastric lavage & biopsy specimen, immunofluorescence, gram stain	Itraconazole 200 mg twice a day x 2 weeks
Alvarez et al. [6]	24 years old	Male	Tanzania	None	3 months of dry cough, fatigue, fever, papulonodular skin lesions, right pleural effusion, weight loss	Chest X ray, punch biopsy of skin with PAS stain, bronchial washings, sputum	Itraconazle 200 mg daily x 6 months
Bennie Ho et al. [9]	39 years old	Male	Southern Saskatchewan	None	Persistent fever, night sweats, left foot arthritis, left foot petechial rash, right hip pain, urinary obstruction	CT chest, MRI foot, joint aspiration with culture & DNA probe, urine fungal culture	Itraconazole 200 mg thrice a day x 3 days followed by 200 mg twice a day x 2 weeks
Arnett et al. [10]	30 years old	Male	Hawaii, USA	San Diego, CA, USA	Worsening non-productive cough, recurrent fevers, weight loss, hematuria	Chest X ray, bronchoscopy, fungal cultures of bronchoalveolar lavage, urine & serum Blastomyces antigen tests	Itraconazole 200 mg twice daily 6 month course
Arnett et al. [10]	21 years old	Male	Hawaii, USA	Iraq	Chronic lower back pain, 2 weeks of non-productive cough, runny nose	CT chest, video-assisted thoroscopic surgery, fungal cultures	None due to absence of pulmonary symptoms
Randhawa et al. [11]	27 years old	Female	India	Minneapolis, Chicago, California, USA	Episodic dry cough, low grade fever, subcutaneous nodules on the anterior abdominal wall	Chest X ray, CT chest, bronchoscopy, excision biopsy of subcutaneous nodule, fungal cultures, DNA extraction and amplification of B. Dermatitidis	Itraconazole 200 mg twice a day x 5 months
Anderson et al. [12]	73 years old	Male	Not specified	Not specified	Asymptomatic, pulmonary nodule	PET chest, CT guided lung biopsy, fungal cultures	Itraconazole 200 mg thrice a day x 2 days then twice a day x 2 months
Anderson et al. [12]	66 years old	Male	Not specified	Not specified	Asymptomatic, weight and appetite loss, pulmonary nodule	Chest X ray, CT chest, bronchoscopy, fungal cultures, urine Blastomyces antigen	Itraconazole 200 mg thrice a day then twice a day x 8 months
Anderson et al. [12]	76 years old	Male	Not specified	Not specified	Asymptomatic, pulmonary nodule	Chest X ray, CT chest, PET scan chest, left thoracotomy & wedge resection, lung biopsy, fungal cultures	Itraconazole 200 mg daily then 400 mg fluconazole daily
Anderson et al. [12]	58 years old	Male	Not specified	Not specified	Asymptomatic, pulmonary nodules	Chest X ray, CT chest, CT guided needle biopsy with fungal cultures	Itraconazole 400 mg daily x 6 months
Anderson et al. [12]	43 years old	Male	Not specified	Not specified	Leg swelling, abdominal pain, shortness of breath, pulmonary nodule	Chest X ray, CT chest, wedge resection, lung biopsy and fungal cultures	Itraconazole 200 mg twice a day x 6 months
Slomka et al. [7]	41 years old	Female	Central Maryland, USA	None	New onset tonic-clonic seizures, weakness of all for extremities	MRI Brain: rim enhancing lesion, brain biopsy and fungal cultures	Oral voriconazole x 3 weeks followed by surgical resection of abscess on day 65, IV iyposomal amphotericin B for 1 week post surgery, high does fluconazole 800 mg x 12 months & dexamethasone till day 79

## Conclusions

To conclude, our case represents the importance of including a differential of fungal pneumonia, besides the most common bacterial and viral pathogens. Even though the patient traveled to a traditionally non-endemic place of *Blastomyces dermatitidis*, a case like this of refractory pneumonia should raise concern for more extensive testing beyond the regular work up. A timely diagnosis can lead to a faster recovery and prevent the chance for potential complications from unnecessary treatment and the disease itself per se.
